# Knowledge, attitudes and practices on antimicrobial resistance among pharmacy personnel and nurses at a tertiary hospital in Ndola, Zambia: implications for antimicrobial stewardship programmes

**DOI:** 10.1093/jacamr/dlac107

**Published:** 2022-10-10

**Authors:** Nanji Tembo, Steward Mudenda, Michelo Banda, Mwitwa Chileshe, Scott Matafwali

**Affiliations:** Department of Clinical Sciences, Copperbelt University, School of Medicine, Ndola, Zambia; Department of Pharmacy, University of Zambia, School of Health Sciences, Lusaka, Zambia; Department of Pharmacy, University of Zambia, School of Health Sciences, Lusaka, Zambia; Department of Pharmacology, Eden University, School of Pharmacy, Lusaka, Zambia; Clinical Research Department, Faculty of Infectious and Tropical Diseases, London School of Hygiene & Tropical Medicine, London, UK

## Abstract

**Introduction:**

Antimicrobial resistance (AMR) is a global public health problem that has led to increased morbidity and mortality, especially in low- and middle-income countries such as Zambia. This study evaluated AMR knowledge, attitudes and practices among pharmacy personnel and nurses at Ndola Teaching Hospital, Zambia’s second-largest hospital.

**Methods:**

A descriptive cross-sectional study was conducted among 263 participants using a structured questionnaire. Data analysis was performed with IBM SPSS version 23.0. All statistical tests were conducted at a 95% confidence level. Univariate analysis was used to determine differences in knowledge, attitudes and practices on AMR between pharmacy personnel and nurses.

**Results:**

Of the 263 participants, 225 (85.6%) were nurses and 38 (14.4%) were pharmacy personnel. Compared with nurses, pharmacy personnel had better knowledge of the spread of resistant bacteria from one person to another (*P* = 0.001) and the use of antibiotics in livestock as a contributing factor to AMR (*P* = 0.01). Pharmacy personnel had better attitudes towards AMR as a public health problem (*P* = 0.001) and the use of antibiotics in livestock as a source of resistant pathogens (*P* = 001). Lastly, more pharmacy personnel than nurses participated in awareness campaigns (*P* = 0.029), continued professional development (*P* = 0.001) and courses on the use of antibiotics and AMR (*P* = 0.028).

**Conclusions:**

The study showed that most participants had adequate knowledge, a positive attitude and good practices towards AMR. Significant differences in knowledge, attitudes and practices were observed between pharmacy personnel and nurses in AMR, highlighting a need for increased educational programmes for these healthcare personnel.

## Introduction

The emerging occurrence of antimicrobial resistance (AMR) is a serious public health problem and threatens to undermine the great strides made in managing infectious diseases.^1^ It has been estimated that in 2019 alone, 4.95 million deaths were associated with AMR, and more than a million people died as a direct result of antibiotic resistance.^2^ Annually, drug-resistant infections, such as MRSA and MDR-TB, contribute to the deaths of thousands, and a review predicted that if left unchecked, 10 million people will die due to AMR by 2050.^1,3^ Concerns are also growing that the COVID-19 pandemic will exacerbate the threat of AMR due to the direct or indirect consequences of pandemic responses.^1^ Although AMR is a challenge worldwide, it disproportionally affects low- and middle-income countries (LMICs) compared with high-income countries due to the high incidence of infectious diseases in these areas.^3,4^

Several AMR drivers include the misuse and overuse of antimicrobials, the lack of access to clean water and hygiene for humans and animals, and poor prevention and control of infection and disease in health facilities and farms.^1^ Besides, the misuse of antimicrobials in animal husbandry has also contributed to the development of AMR.^5–8^ The lack of access to quality-assured medicines also contributes to AMR because patients are exposed to ineffective, substandard and falsified medicines.^9^ Other drivers include lack of awareness and knowledge regarding AMR and lack of enforcement of legislation. Zambia, like other LMICs, has reported high levels of irrational antimicrobial prescribing and dispensing.^10–13^ There are also high rates of self-medication, purchase of antibiotics without prescription and non-compliance in Zambia, which can contribute to AMR.^14,15^ In a study done to assess the non-prescription sale and dispensing of antibiotics in community pharmacies in Lusaka, Zambia, 97% of pharmacy personnel indicated that clients frequently requested antibiotics without a prescription, and 100% indicated that clients had purchased antibiotics without a prescription.^13^

Knowledge and understanding of information related to antimicrobial activity and its relationship with resistance can be valuable in establishing programmes for the use of antibiotics.^16,17^ An example of these programmes includes establishing antimicrobial stewardship (AMS) programmes in health facilities, a key strategy that promotes rational use of antimicrobials and has shown significant benefit.^17^ Healthcare workers (HCWs), such as pharmacy personnel and nurses, have an important role in overcoming the challenge of AMR since they are responsible for providing adequate healthcare through the distribution of medicines and the provision of information on the use of prescribed medicines.^18,19^ In Zambia, pharmacists are not prescribers,; however, pharmacists have important roles such as consumer education and ensuring rational use of antibiotics in hospitals, which are key components in the fight against AMR.^17^ It is estimated that over 50% of antimicrobial use (AMU) within the hospital setting is inappropriate and associated with poor patient outcomes and the development of AMR.^20,21^ One of the major components in AMS is that ‘the right antimicrobial medicine at the right dose should be administered at the right time for the right duration’; nurses, having a persistent presence within in-patient care settings, are in the ideal position to ensure correct use of antimicrobial agents.^21^

The success of AMS programmes depends on several issues, including: multidisciplinary approach; HCW knowledge, attitudes and practices; health structure challenges; lack of implementation of policies; and inadequate surveillance systems and governance.^22–25^ To address the threat of AMR and implement successful AMS programmes, HCWs must have the right information. Despite efforts to establish AMS programmes, there is little information on the knowledge, attitudes and practices regarding AMR among HCWs at the second largest hospital in Zambia. Therefore, this study assessed the knowledge, attitudes and practices of pharmacy personnel and nurses at Ndola Teaching Hospital (NTH) in Ndola, Zambia.

## Methods

### Study design, site and population

This cross-sectional study was conducted at NTH in the Ndola district in the Copperbelt province from January 2022 to April 2022. The hospital was selected as no studies have been conducted on assessing knowledge, attitudes and practices on antibiotic resistance among pharmacy personnel and nurses. Additionally, the hospital is a large referral hospital for Copperbelt, Luapula and North-western provinces of Zambia with a bed count of about 1000.^26^ The hospital provides both primary outpatient and inpatient tertiary care services. The hospital was also suitable because more pharmacy personnel and nurses were available to be surveyed. This study targeted pharmacy personnel and nurses involved in dispensing, administering and counselling patients on how to use antibiotics. To be eligible, the pharmacy personnel and nurses were to be employees at NTH and provided consent to participate in the study. All pharmacy personnel and nurses on leave during data collection were not included in the study. The enrolled and registered nurses represented all wards, including inpatient, outpatient, adult, medical and surgical. Additionally, pharmacy and nursing students were excluded from the study.

### Sample size estimation and sampling technique

The sample sizes of pharmacy personnel and nurses were independently determined using Yamane’s formula; $n=\frac{N}{1+N{\left(e\right)}^{2}}$.^27^ The study site comprised 513 nurses and 42 pharmacy personnel. Using a confidence level of 95%, a margin of error of 5%, a 10% incomplete or non-response or loss to follow-up was considered, giving a minimum of 253 required participants. After sample size estimation, the participants were selected using convenient sampling because they worked in shifts during the COVID-19 pandemic. Data collection was done in the morning, afternoon, evening and night shifts to increase the chances of sampling all potential participants. Therefore, the obtained enrolled participants in the study were representative of all pharmacy and nursing personnel working at NTH. Additionally, we distributed 265 questionnaires to 230 nurses and 35 pharmacy personnel.

### Data collection tool

Questionnaires were distributed among pharmacy personnel and nurses willing to participate in the study and were in line with the inclusion criteria. This questionnaire included questions about knowledge about AMR and its causes (six questions), attitudes toward AMR (six questions), practices towards the control of AMR (six questions) and sociodemographic characteristics of participants. A correct response was assigned a score of 1, while an incorrect response was assigned a score of 0, resulting in a maximum score of 6 for knowledge, attitudes and practices. Based on a recent study, knowledge, attitudes and practices was assessed as good if scores were ≥80%, moderate if scores were between 60% and 79%, or poor if scores were less than 60%.^28^ The questionnaire was adapted from similar studies undertaken by Mitwali.^29^ The questionnaire was piloted for consistency, length and relevance of the questions among five physicians and five intern pharmacists at NTH. The obtained data from the pilot study were not used in the final analysis of the main study findings. The data were collected by two data collectors trained in administering the questionnaire while adhering to the COVID-19 prevention measures. Responding to the questionnaire took between 15 and 30 min.

### Data analysis

The collected data were entered into Microsoft Excel and exported into IBM SPSS version 23.0 for analysis. The analysed data was presented in tables. All statistical tests were conducted at a 95% confidence level. Univariate analysis was used to determine the knowledge, attitudes and practices differences between pharmacy personnel and nurses on AMR.

### Ethics approval

Ethical clearance for the study was sought from the Tropical Diseases Research Centre (TDRC) with protocol ID of TRC/C4/11/2021. Additional clearance from the National Health Research Authority (NHRA) was obtained with an approval number of NHRA000011/06/01/2022. After ethical approval, institutional clearance was obtained from the NTH administration. Informed consent was obtained from the participants, and privacy and confidentiality were assured regarding the collected information.

## Results

### Sociodemographic characteristics of participants

Table 1 shows a total number of 263 health workers enrolled in the study. Most participants were female (82.1%) and aged above 30 years (56.7%). The study participants were predominantly nurses (85.6%). Most participants had a Bachelor’s degree (61.2%) and had worked for 1 to 5 years (45.2%).

**Table 1.** Sociodemographic characteristics of participants (*n* = 263)

Variable	Category	Frequency (*n*, %) for nurses	Frequency (*n*, %) for pharmacists	*P* value
Gender	Female	195 (90.3)	21 (9.7)	0.001
	Male	30 (63.8)	17 (36.2)	
Age (years)	≥30	143 (96)	6 (4.0)	0.001
	<30	82 (71.9)	32 (28.1)	
Qualification	Certificate	12 (100)	0 (0.0)	0.001
	Diploma	157 (97.5)	4 (2.5)	
	Bachelors’ degree	56 (62.9)	33 (37.1)	
	Master’s degree	0 (0.0)	1 (100)	
Work experience (years)	<1	0 (0.0)	5 (100)	0.001
	1–5	96 (80.7)	23 (19.3)	
	6–10	34 (85)	6 (15)	
	>10	95 (96)	4 (4.0)	

### Knowledge, attitudes and practices of participants on AMR

Table 2 shows that most participants knew that it was wrong to use antibiotics left by someone (97%) and that infections caused by resistant bacteria are difficult to treat (85.9%). Most (54.4%) participants knew that AMR was a public health problem. Conversely, the majority (60.1%) of the participants never participated in campaigns promoting optimal antibiotic use. Pharmacy personnel had better attitudes concerning AMR as a global issue and livestock as a source of new resistant pathogens. They also had better practices regarding campaigns, continuous professional development (CPD) and taking courses in AMR.

**Table 2.** Knowledge, attitudes and practices of participants on AMR

Variable	Questions	Professional Category	Responses		*P* value
			Yes, *n*	No, *n*	
Knowledge questions	1. It is okay for someone to use antibiotics that were administered to a friend or family member, as long as they were used to treat the same illness (correct answer = ‘No’)	Pharmacy personnel	2	36	0.325
		Nurse	6	219	
	2. Many infections are increasingly resistant to antibiotic treatment (correct answer = ‘Yes’)	Pharmacy personnel	28	10	0.273
		Nurse	151	74	
	3. Infections caused by antibiotic-resistant bacteria are difficult or impossible to treat (correct answer = ‘Yes’)	Pharmacy personnel	33	5	0.548
		Nurse	193	32	
	4. Antibiotic resistance is an issue in other countries but not in ours (correct answer = ‘No’)	Pharmacy personnel	3	35	0.130
		Nurse	37	188	
	5. Bacteria that are resistant to antibiotics can be spread from person to person (correct answer = ‘Yes’)	Pharmacy personnel	33	5	**0**.**001**
		Nurse	105	120	
	6. The risk of antimicrobial resistance increases in individuals that consume livestock that is treated with antimicrobials (correct answer = ‘Yes’)	Pharmacy personnel	18	20	**0**.**010**
		Nurse	60	165	
Attitude questions	1. Antibiotic resistance is a major public health problem in our country (correct answer = ‘Yes’)	Pharmacy personnel	34	4	**0**.**001**
		Nurse	109	116	
	2. The fact that one is taking an antibiotic increases the chances of developing resistance (correct answer = ‘Yes’)	Pharmacy personnel	15	23	0.330
		Nurse	101	124	
	3. New antibiotic development can solve antibiotic resistance problems (correct answer = ‘Yes’)	Pharmacy personnel	20	18	0.435
		Nurse	125	100	
	4. The use of antibiotics in livestock animals is an important cause of the appearance of new resistance to pathogenic agents in humans (correct answer = ‘Yes’)	Pharmacy personnel	26	12	**0**.**001**
		Nurse	56	169	
	5. In all cases where antibiotics are dispensed, it is important that patients are advised about complying with the treatment (correct answer = ‘Yes’)	Pharmacy personnel	37	1	0.094
		Nurse	201	24	
	6. There is need to establish a course on the ‘rational use of antibiotics’ (correct answer = ‘Yes’)	Pharmacy personnel	36	2	0.076
		Nurse	191	34	
Practice questions	1. I educate people on the use of antibiotics and resistance related issues whenever I can (correct answer = ‘Yes’)	Pharmacy personnel	35	3	0.372
		Nurse	213	12	
	2. I participate in antibiotic awareness campaigns to promote the optimal use of antibiotics (correct answer = ‘Yes’)	Pharmacy personnel	21	17	**0**.**029**
		Nurse	84	141	
	3. I have attended Continuing Professional Development (CPD) education on antibiotic use and resistance topics (correct answer = ‘Yes’)	Pharmacy personnel	22	16	**0**.**001**
		Nurse	54	171	
	4. I have taken courses to improve my knowledge of antimicrobial resistance and antibiotic use (correct answer = ‘Yes’)	Pharmacy personnel	17	21	**0**.**028**
		Nurse	62	163	
	5. I follow the standard treatment guidelines when dealing with infectious diseases (correct answer = ‘Yes’)	Pharmacy personnel	37	1	0.094
		Nurse	201	24	
	6. I adhere to infection control guidelines established at our facility (correct answer = ‘Yes’)	Pharmacy personnel	36	2	0.539
		Nurse	215	10	

Bold values indicate results that are statistically significant.

### Difference in knowledge, attitudes and practices regarding AMU and AMR among nurses (n = 225)

Most diploma and degree-holder nurses did not know individuals who eat livestock treated with antibiotics are at high risk of developing AMR (Table 3). Additionally, most diploma holders had not participated in AMR campaigns, or CPD, and never took a course to improve their knowledge and practices on AMR (*P* = 0.001).

**Table 3.** Difference in knowledge, attitudes and practices regarding AMU and AMR among nurses (*n* = 225)

Questions	Qualifications	Responses		*P* value
		Yes	No	
Bacteria that are resistant to antibiotics can be spread from person to person	Certificate	6	6	0.176
	Diploma	67	90	
	Degree	32	24	
The risk of antimicrobial resistance increases in individuals that consume livestock that is treated with antimicrobials	Certificate	6	6	**0.013**
	Diploma	46	111	
	Degree	8	48	
Antibiotic resistance is a major public health problem in our country	Certificate	6	6	0.294
	Diploma	71	86	
	Degree	32	24	
The use of antibiotics in livestock animals is an important cause of the appearance of new resistance to pathogenic agents in humans	Certificate	6	6	0.073
	Diploma	34	123	
	Degree	16	40	
I participate in antibiotic awareness campaigns to promote the optimal use of antibiotics	Certificate	12	0	**0**.**001**
	Diploma	44	113	
	Degree	28	28	
I have attended Continuing Professional Development (CPD) education on antibiotic use and resistance topics	Certificate	6	6	**0**.**001**
	Diploma	26	131	
	Degree	22	34	
I have taken courses to improve my knowledge of antimicrobial resistance and antibiotic use	Certificate	0	12	**0**.**001**
	Diploma	28	129	
	Degree	34	22	

Bold values indicate results that are statistically significant.

### Overall knowledge, attitudes and practices regarding AMR among study participants

Overall, the participants scored 70%, 60% and 64% on knowledge, attitudes and practices (moderate knowledge, attitudes and practices) questions regarding AMR (Table 4).

**Table 4.** Overall knowledge, attitudes and practices regarding AMR among participants

Variable	Scores (%)
Knowledge	70
Attitudes	60
Practices	64

## Discussion

This study aimed to assess pharmacy and nursing personnel’s knowledge, attitudes and practices regarding AMR at a tertiary hospital in Ndola, Zambia. This study found a moderate knowledge, attitudes and practices score on AMR among the participants. Most participants knew that sharing antibiotics with friends or family is wrong practice. Such practices have been reported to be a form of self-medication and a contributor to AMR.^30,31^ In Zambia, the government in 2017 launched a multisectoral national action plan on AMR, with one aim to enhance knowledge about antibiotic use and resistance.^32^ One of the methods used to achieve this was the launch of the annual World Antibiotic Awareness Week (WAAW). Several key messages were delivered during this campaign, one of which was to discourage the sharing of antimicrobials.^33^

The current study found that most participants, especially diploma-holder nurses, did not know that antibiotic-resistant bacteria can spread from person to person. This starkly contrasts with a multinational EU/European Economic Area (EEA) study in which almost 90% of HCWs were aware of this.^34^ The high proportion of HCWs who were knowledgeable about this topic area was attributed to European Antibiotic Awareness Day (EAAD), an annual initiative targeting both the public and health workers, whose purpose is to raise awareness about the need for the judicious use of antibiotics. Future WAAW or similar programmes in Zambia can address this important subject.

Interestingly, most pharmacists, compared with nurses, were aware that AMR is a public health problem. These findings align with other studies that reported that pharmacists are aware of AMR as a public health issue. Almost half of the participants (45.6%) did not agree with ‘Antibiotic resistance is a major public health problem in our country’; this is in contrast to what was found in Zimbabwe, where 83% of HCWs agreed.^35^ Nurses made up the majority of health workers (116/120, *P* = 0.001) who disagreed with this statement. Interestingly, a large proportion of pharmacy personnel and nurses (84.8%) agreed that antibiotic resistance is a problem in Zambia (knowledge item 4), with no statistically significant differences in agreement between pharmacy personnel and nurses. The survey on antibiotic use and resistance conducted among health workers in South Africa also had similar findings; the study revealed that health workers thought antibiotic resistance was a global and national problem, with a lower proportion of nurses compared with pharmacists who were aware of this.^36^ This could mean that nurses thought antibiotic resistance was an issue but not significant or serious enough to be considered a public health problem. One way to address this negative attitude would be to include this message in future WAAW campaigns. Once healthcare professionals understand the gravity of antibiotic resistance, they are more likely to handle them with care and encourage their patients to do the same.

Our study found that most participants did not know that taking antibiotics increases the risk of developing bacterial AMR. These findings are similar to those obtained in other LMICs.^37,38^ This attitude problem requires urgent attention as it may promote unnecessary use of antibiotics with the development of resistant pathogens. A recent evaluation of pathogen antibiotic susceptibility conducted at the same hospital has shown the presence of resistant bacteria in isolates from both inpatients and outpatients, ^39^ indicating the need to scale up interventions to prevent unnecessary antibiotic use. The EU/EAA multinational antibiotic resistance survey earlier highlighted that more than 70% of HCWs were aware of this due to the EAAD that contained this message. Future WAAWs could tailor messages to fit local attitudes like this one.

Most of our study participants, especially diploma and degree-holder nurses, did not believe that the use of antibiotics in livestock animals contributed to the development of new antibiotic-resistant pathogens in humans. This is directly related to their lack of knowledge, as 70.3% believed the intake of antimicrobial-treated livestock could not contribute to resistance. Other studies have reported similar findings.^40,41^ This phenomenon is worrying because of the increasing global trends of cross-over use of human and livestock antibiotics often obtained from hospitals and community pharmacies.^7,42^

In the questions on knowledge and attitudes, nurses made up the majority of those who answered incorrectly and was statistically different from pharmacists (*P* = 0.01 for knowledge, *P* = 0.001 for attitude). The findings are similar to those of a survey among health workers in South Africa that aimed to investigate HCWs’ knowledge, attitudes and practices about antibiotic use and resistance. In the said study, significantly more nurses than pharmacists did not know that the use of antibiotics in animals contributes to resistance and thus did not think that reducing AMU in animals could reduce AMR.^36^ Previous efforts by the Zambian government have educated agriculture and veterinarian personnel about the contribution of antibiotic use in plants and livestock to AMR.^33^ However, this message must be delivered to HCWs so they can be fully equipped to deal with issues related to AMR.

Most pharmacists and nurses promoted positive practices regarding antibiotic use and resistance in three practice areas: the education of the public about antibiotic use and resistance, use of the standard treatment guidelines when dealing with infectious diseases, and adherence to the facility infection control guidelines. Most participants in our study responded that they participated in educating the public about the use of antibiotics and resistance. However, the majority of them, especially diploma and degree-holder nurses, did not participate in antibiotic awareness campaigns and did not take any CPD education or other courses that could improve their knowledge, attitudes and practices regarding antibiotic use and resistance. This is similar to the study conducted in South Africa, where most pharmacists and nurses had not attended workshops or training in AMS.^36^ There is a need to improve the knowledge, attitudes and practices of HCWs on AMR by promoting the establishment of AMS programmes in healthcare facilities and encouraging pharmacists and nurses to enrol for CPD education on AMU and AMR.^43,44^

In the current study, a statistically higher proportion of pharmacy personnel were more aware of the potential spread of antibiotic-resistant organisms from person to person and of the increased risk of AMR in individuals consuming antibiotic-treated livestock. Furthermore, a statistically more significant proportion of pharmacy personnel thought antibiotic resistance was a public health problem and that antibiotic use in livestock was an important source of developing new antibiotic-resistant strains. This could be due to the statistically higher proportion of pharmacy personnel compared with nurses who participated in campaigns and took CPD education and other courses regarding antibiotic use and resistance. Therefore, educational activities during campaigns could be targeted toward nurses, and CPD programmes or workshops could be targeted to pharmacists and nurses to enhance their knowledge, attitudes and practices towards judicious use of antibiotics.

Overall, our study found a moderate knowledge, attitudes and practices score regarding AMR among the participants. These findings identify gaps regarding AMR among pharmacy and nursing personnel working at NTH. These findings are similar to what was reported in Nepal, where HCWs had moderate knowledge, attitudes and practices concerning AMR.^28^ Similar findings were reported in South Africa, in which gaps in knowledge, attitudes and practices regarding AMR were identified among pharmacists and nurses.^36^ The current findings and those reported from other studies suggest the need for increasing training and educational activities concerning AMR. Alongside this, AMS programmes should be strengthened in all areas of clinical practice. An educational interventional strategy has been reported to be effective in improving knowledge, attitudes and practices regarding AMR.^45^ This may increase the awareness of pharmacy and nursing personnel concerning AMR, which can in turn improve their knowledge, attitudes and practices.

### Limitations

We acknowledge that only nurses and pharmacists at one large referral hospital were surveyed in this study. Our findings may not be generalized to all HCWs in Zambia, as only a section of HCWs was considered, but we are confident that our findings are useful in strengthening the current AMS programmes and developing new ones. Due to limited resources, other HCWs were not surveyed. However, key HCWs, nurses who represent the highest number of HCWs and perform prescribing functions, have been represented. Despite these limitations, our findings provide valuable baseline data for future studies and programmes.

### Recommendations

A previous study showed that most campaign messages were similar in high-income and low-income countries despite differences in antibiotic use and resistance in these regions.^46^ To overcome this, the impact of campaigns such as WAAW must be assessed through methods such as surveys among health workers, and such surveys can highlight the knowledge, attitudes and practices gaps that can be targeted in future campaigns. The Zambian government plans to take an evidence-based approach to design communications regarding AMR following behavioural studies amongst professions.^32^ It could extend this to selecting key messages where knowledge, attitudes and practices were poor following surveys. This strategy is also described by the EU/EEA study highlighted earlier.^34^

### Conclusions

The study showed that most participants had moderate knowledge, positive attitudes and practices toward AMR. However, the study revealed significant differences in the knowledge, attitudes and practices between pharmacy personnel and nurses on AMR. Pharmacy personnel demonstrated higher knowledge levels, more positive attitudes and better practices towards AMR than nurses. These gaps may significantly affect the rational use of antimicrobials. The differences and moderate knowledge, attitudes and practices regarding AMR identified in this study further indicate the need for increased educational and sensitization programmes across the pharmacy and nursing professions.
